# Mapping EORTC QLQ-C30 onto EQ-5D for the assessment of cancer patients

**DOI:** 10.1186/1477-7525-10-151

**Published:** 2012-12-17

**Authors:** Seon Ha Kim, Min-Woo Jo, Hwa-Jung Kim, Jin-Hee Ahn

**Affiliations:** 1 Department of Preventive Medicine, University of Ulsan College of Medicine, 86, Asanbyeongwon-gil, Songpa-gu, Seoul, 138-736, Korea; 2 Department of Clinical Epidemiology and Biostatistics, Asan Medical Center, 86, Asanbyeongwon-gil, Songpa-gu, Seoul, 138-736, Korea; 3 Division of Oncology, Asan Medical Center, University of Ulsan College of Medicine, 86, Asanbyeongwon-gil, Songpa-gu, Seoul, 138-736, Korea

**Keywords:** EQ-5D, EORTC QLQ-C30, Cancer, Mapping, Quality of life

## Abstract

**Background:**

The European Organisation for Research and Treatment of Cancer Quality of Life Questionnaire Core 30 (EORTC QLQ-C30) is the instrument most frequently used to measure quality of life in cancer patients, whereas the EQ-5D is widely used to measure and evaluate general health status. Although the EORTC QLQ-C30 has been mapped to EQ-5D utilities, those studies were limited to patients with a single type of cancer. The present study aimed to develop a mapping relationship between the EORTC QLQ-C30 and EQ-5D-based utility values at the individual level.

**Methods:**

The model was derived using patients with different types of cancer who were receiving chemotherapy. The external validation set comprised outpatients with colon cancer. Ordinary least squares regression was used to estimate the EQ-5D index from the EORTC QLQ-C30 results. The predictability, goodness of fit, and signs of the estimated coefficients of the model were assessed. Predictive ability was determined by calculating the mean absolute error, the estimated proportions with absolute errors > 0.05 and > 0.1, and the root-mean-squared error (RMSE).

**Results:**

A model that included global health, physical, role, emotional functions, and pain was optimal, with a mean absolute error of 0.069 and an RMSE of 0.095 (normalized RMSE, 8.1%). The explanatory power of this model was 51.6%. The mean absolute error was higher for modeled patients in poor health.

**Conclusions:**

This mapping algorithm enabled the EORTC QLQ-C30 to be converted to the EQ-5D utility index to assess cancer patients in Korea.

## Background

In addition to assessing of clinical efficacy, appraisals of new healthcare technology need to assess cost-effectiveness. Cost-utility analysis is frequently used for economic evaluation, with outcomes evaluated in terms of quality-adjusted life years, a measure that combines both the length and quality of life. Utilities are preference-based and derived from each individual, either directly using valuation techniques such as standard gamble, time trade-off, or the use of a rating scale, or indirectly using generic health-related quality of life (HRQoL) measures, such as the Health Utility Index [1,2], the EuroQol 5D (EQ-5D), [3] or the Short Form 6D [4]. Scoring algorithms have been developed for all of these measures, which provide community-based health utility estimates [5].

HRQoL is often used as a secondary endpoint in cancer trials. Studies measuring patient quality of life often prefer disease-specific instruments over generic instruments. The former focus on particular health problems and tend to be more sensitive to clinically important differences [6]. They do not, however, include utility scoring systems. Therefore, the development of a tool that can map disease-specific measures onto preference-based measures may also generate weighted utilities.

The European Organisation for Research and Treatment of Cancer Quality of Life Questionnaire Core 30 (EORTC QLQ-C30) is the instrument most frequently used to measure the quality of life of cancer patients [7]. The Korean version of the EORTC QLQ-C30 has been validated for use in Korean cancer patients [8]. Although the EORTC-8D, a preference-based measure derived from the EORTC QLQ-C30, was recently introduced [9], the results obtained from these questionnaires cannot be compared with the results of questionnaires based on other disease areas because the EORTC QLQ-C30 is a cancer-specific instrument. Moreover, to our knowledge, no valuation set for the EORTC-8D has yet been developed in Korea. The EQ-5D, an instrument widely used to measure and evaluate general health status, can also be used to assign preference values to these health states. Population tariffs using the EQ-5D have been developed in several countries, including Korea [10,11]. Although the EORTC QLQ-C30 has been mapped onto EQ-5D utilities [5,12-14], those studies were limited to patients with a single type of cancer.

The purpose of this study was to develop a mapping relationship between the EORTC QLQ-C30 and EQ-5D-based utility values at the individual level for patients with a wide range of cancers.

## Methods

### Data set and instruments

We used two data sets to formulate the mapping algorithm for the EORTC QLQ-C30 and the EQ-5D. The derivation set comprised 893 patients with different types of cancer [15], whereas the external validation set comprised 123 patients with colon cancer [16]. The patients in these two studies were independent of each other, but were recruited at the same cancer center.

The EQ-5D comprises five dimensions that measure general health status: mobility, self-care, usual activities, pain/discomfort, and anxiety/depression, with each dimension having three levels. Thus, the EQ-5D provides a simple descriptive profile and a single utility index of health status, which can be used in the clinical and economic evaluation of healthcare, as well as for population health surveys [10]. The EQ-5D index for use in Korea was calculated using an algorithm [11], with possible scores ranging from −0.171 to 1.0, with 1.0 indicating full health (11111 state) and 0.0 denoting death.

The EORTC QLQ-C30 is an integrated system that assesses the HRQoL of cancer patients. It includes five functional scales (physical, role, emotional, cognitive, and social), three symptom scales (fatigue, nausea or vomiting, and pain), global health status, and six single items (dyspnea, insomnia, appetite loss, constipation, diarrhea, and financial difficulties) [7]. All of these scales and items were linearly transformed from 0 to 100 according to the EORTC QLQ-C30 scoring rules [17]. High scores on the functional scales indicate a high level of functioning and high scores on the global health status indicate a high quality of life; by contrast, high scores on the symptom scales/items indicate high levels of health problems [17].

### Analysis

Ordinary least squares (OLS) regression was used to estimate the EQ-5D index from the EORTC QLQ-C30. The dependent variable was the EQ-5D index, and the explanatory variables were the EORTC QLQ-C30 scale and item scores. All variables were treated as continuous variables. The full model, which included the scores for all scales and items in the EORTC QLQ-C30, was explored and another model was developed using backward elimination with a significance level of 0.1 from the full model.

The relationships between the observed and predicted values were assessed visually. The performance of each model was evaluated by determining its predictability, goodness of fit, and the signs of the estimated coefficients. The purpose of a mapping function is to predict health utility values in other data sets; therefore, the model was assessed according to the accuracy of its predictions [18]. Predictive ability was determined by calculating the mean absolute error (MAE), the estimated proportions with absolute errors > 0.05 and >0.1, and the root-mean-squared error (RMSE). The MAE is the average of the absolute differences between the observed and predicted values, and the RMSE is the root of the average of the squared differences. RMSE can also be reported as a percentage of the scale size (i.e., 1.171, the range of the EQ-5D-based utility according to the Korean algorithm [11]), referred to as the normalized RMSE [19]. Smaller MAE and RMSE values indicate better model performance. The important aspect of the mapping was the estimated group mean and its variance, rather than individual estimated utilities. To determine whether errors were affected by disease severity, both the highest and the lowest EQ-5D index quartile groups of the derivation and validation sets were evaluated separately. The overall equality of the coefficients of the good health group and other groups was tested using the likelihood ratio test. In addition, the adjusted R^2^ values and the signs of the estimated coefficients were calculated. The sign of the functional scales was expected to be positive, while that of the symptom scales/items was expected to be negative.

Statistical analyses were performed using SAS software (ver. 9.1; SAS Institute Inc., Cary, NC). P<0.05 was considered statistically significant.

## Results

The derivation set included patients with 28 different types of cancer (Table 1). Breast cancer was the most common (32.9%), followed by colorectal cancer (20.0%). Table 2 presents the descriptive statistics for the EQ-5D index and the EORTC QLQ-C30 scales of the derivation and validation sets. Patients in the derivation set generally had poorer scores on all scales (except for diarrhea) than patients in the validation set. Differences between scale and item scores were statistically significant, except for “constipation” and “financial difficulty”.

**Table 1:** Distribution of cancer patients in the derivation set

**Type of cancer**	**N**	**(%)**
Breast	291	(32.9)
Colorectal	177	(20.0)
Lung	98	(11.1)
Stomach	89	(10.1)
Pancreas	39	(4.4)
Bone marrow	31	(3.5)
Liver	31	(3.5)
Lymph node	23	(2.6)
Esophagus	20	(2.3)
Gall bladder	14	(1.6)
The others (18 types)	71	(8.0)

**Table 2:** Descriptive statistics of the EQ-5D index and the EORTC QLQ-C30 scales used in the derivation and validation sets

**Variables**	**Derivation set**			**Validation set (n = 123)**	
	**n**	**Mean (SD)**	**Median (IQR)**	**Mean (SD)**	**Median (IQR)**
EQ-5D index	893	0.824 (0.137)	0.854 (0.723–0.907)	0.871 (0.113)	0.87 (0.817–1.000)
EORTC QLQ-C30 functional scales					
Global health status^*^	893	59.8 (21.9)	66.7 (25.0–50.0)	68.2 (22.5)	66.7 (50.0–83.3)
Physical functioning^*^	893	72.1 (18.3)	73.3 (60.0–86.7)	79.2 (16.0)	80.0 (73.3–93.3)
Role functioning^*^	893	68.2 (27.1)	66.7 (50.0–100)	77.2 (21.9)	83.3 (66.7–100)
Emotional functioning^*^	893	70.9 (23.2)	75.0 (58.3–91.7)	79.8 (21.1)	83.3 (66.7–100)
Cognitive functioning^*^	892	76.5 (20.8)	83.3 (66.7–100)	80.5 (19.4)	83.3 (66.7–100)
Social functioning^*^	893	62.8 (28.2)	66.7 (50.0–83.3)	73.2 (23.0)	66.7 (66.7–100)
EORTC QLQ-C30 symptom scales/items					
Fatigue^*^	893	40.6 (22.0)	33.3 (22.2–55.6)	33.0 (19.8)	33.3 (22.0–44.4)
Nausea and vomiting^*^	893	22.5 (25.5)	16.7 (0.0–33.3)	14.9 (21.8)	0.0 (0.0–16.7)
Pain^*^	893	28.8 (26.2)	16.7 (0.0–50.0)	17.1 (21.2)	16.7 (0.0–33.3)
Dyspnea^*^	892	24.4 (26.8)	33.3 (0.0–33.3)	17.9 (21.9)	0.0 (0.0–33.3)
Insomnia^*^	889	31.3 (30.6)	33.3 (0.0–33.3)	19.5 (23.7)	0.0 (0.0–33.3)
Appetite loss^*^	889	31.8 (31.4)	33.3 (0.0–66.7)	18.4 (24.2)	0.0 (0.0–33.3)
Constipation	892	26.1 (29.3)	33.3 (0.0–33.3)	20.9 (26.8)	0.0 (0.0–33.3)
Diarrhea^*^	892	18.7 (26.0)	0.0 (0.0–33.3)	27.4 (28.6)	33.3 (0.0–33.3)
Financial difficulties	892	37.9 (33.3)	33.3 (0.0–66.7)	31.7 (30.1)	33.3 (0.0–66.7)

^*^p < 0.05; Student’s *t* test.

**Figure 1:**
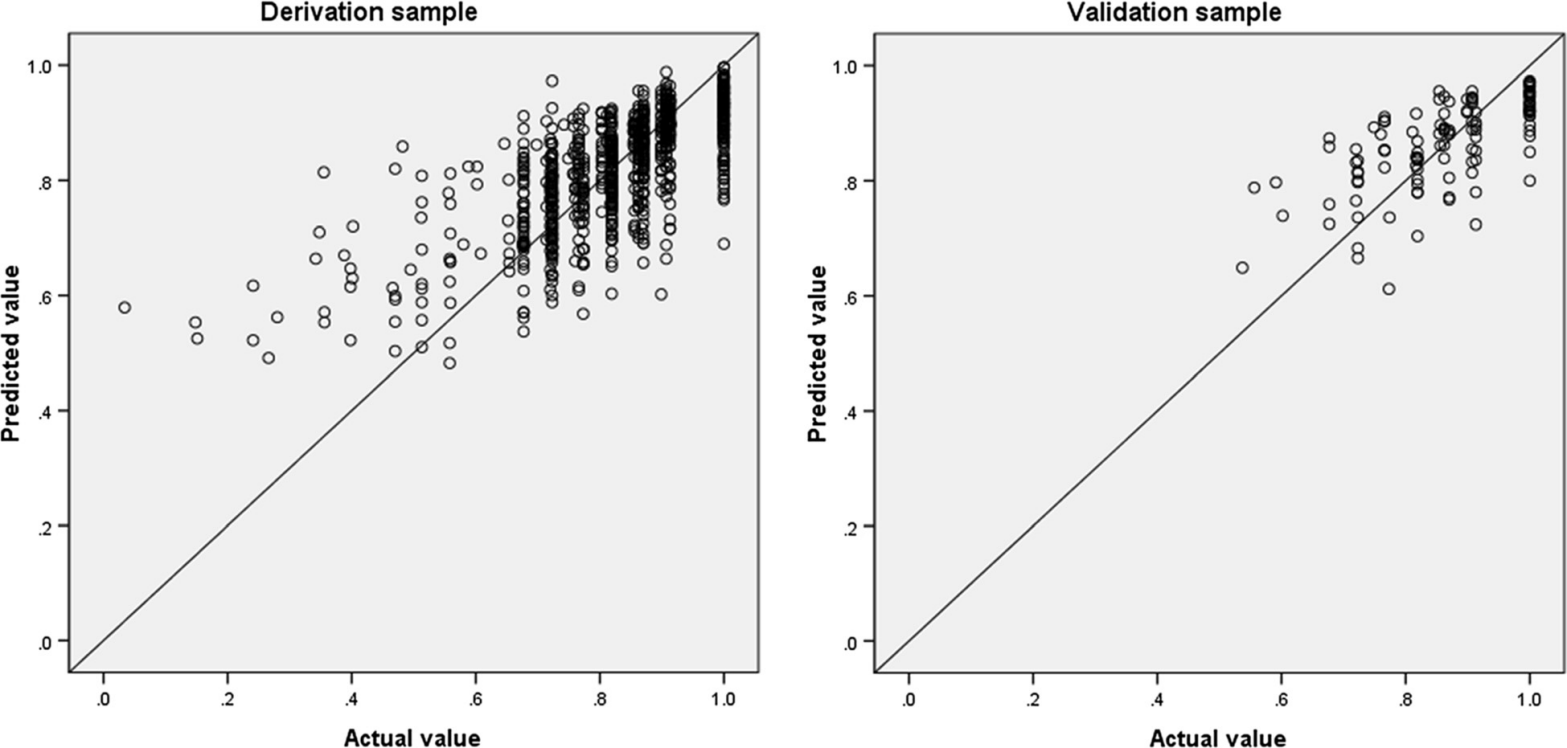
**Scatter plot of predicted values based on Model 2 parameters versus the actual EQ-5D index.** A perfect fit is indicated by the 45° reference line.

The results of the OLS regression analysis of each of the two models are shown in Table 3, and model performance is shown in Table 4. In Model 2 (i.e., the backward elimination model), the five scales were statistically significant; the emotional functioning scale, which had a p value of 0.071 in Model 1, became statistically significant in Model 2, with a p value of 0.01. Physical functioning was the most influential scale in both models (Table 3). The explanatory power of Model 2 was 51.6%. The MAE values of both models were the same: 0.095 for the derivation set and 0.066 for the validation set. In Model 2, the normalized RMSE was 8.1% for the derivation set and 7.2% for the validation set. The proportion of AEs > 0.1 in Model 2 was 23.1% for the derivation set and 24.4% for the validation set. The actual mean value of the EQ-5D index was similar to the predicted EQ-5D indices of both models (Table 4). Figure 1 shows a plot of the predicted value based on Model 2 versus the observed EQ-5D index in both the derivation and validation sets. In both sets, EQ-5D index for values below 0.7 tended to be overestimated, whereas the maximum value of EQ-5D was underestimated.

**Table 3:** Ordinary least squares regression model

	**Model 1^a^**			**Model 2^b^**		
	**β**	**SE**	***p *value**	**β**	**SE**	***p *value**
Intercept	0.53897	0.03507	< 0.0001	0.56317	0.02044	< 0.0001
Global health status	0.00092	0.00018	< 0.0001	0.00097	0.00018	< 0.0001
Physical functioning	0.00223	0.00027	< 0.0001	0.00222	0.00026	< 0.0001
Role functioning	0.00065	0.00019	0.001	0.00067	0.00018	0.0001
Emotional functioning	0.00038	0.00021	0.071	0.00045	0.00017	0.01
Cognitive functioning	0.00015	0.00021	0.474			
Social functioning	0.0002	0.00017	0.234			
Fatigue	0.00042	0.00027	0.111			
Nausea and vomiting	−0.00005	0.00015	0.737			
Pain	−0.00123	0.00017	< 0.0001	−0.00125	0.00016	< 0.0001
Dyspnea	−0.00024	0.00015	0.102			
Insomnia	−0.00009	0.00013	0.494			
Appetite loss	−0.00001	0.00014	0.943			
Constipation	−0.00004	0.00012	0.72			
Diarrhea	0.00005	0.00013	0.72			
Financial difficulties	0.00005	0.00012	0.673			

^a^Model 1 included all functioning and symptom scales and items of the EORTC QLQ-C30 as explanatory variables.

^b^Model 2 applied backward elimination to Model 1.

**Table 4:** Comparison of the performance of Models 1 and 2

		**Model 1^a^**	**Model 2^b^**
Derivation set			
Adjusted R^2^		0.511	0.516
RMSE (% RMSE)		0.095 (8.1)	0.095 (8.1)
MAE (SD)		0.069 (0.065)	0.069 (0.066)
MAE > 0.05(%)		48.7	50.1
MAE > 0.1(%)		22.4	23.1
EQ-5D index	Actual	Predicted	Predicted
Mean (SD)	0.824 (0.137)	0.823 (0.098)	0.824 (0.098)
Validation set			
RMSE (% RMSE)		0.083 (7.1)	0.085 (7.2)
MAE (SD)		0.066 (0.052)	0.066 (0.053)
MAE > 0.05(%)		53.7	49.6
MAE > 0.1(%)		23.6	24.4
EQ-5D index	Actual	Predict	Predict
Mean (SD)	0.871 (0.113)	0.873 (0.083)	0.872 (0.085)

^a^Model 1 included all functioning and symptom scales and items of the EORTC QLQ-C30 as explanatory variables.

^b^Model 2 applied backward elimination to Model 1.

Table 5 shows the model performance for both the derivation and validation sets according to health status when Model 2 was fitted. The MAEs of the lowest quartile group on the EQ-5D for the derivation (≤0.723) and validation (≤0.817) sets were 0.100 and 0.060 respectively, whereas the MAEs of the highest quartile group on the EQ-5D for the derivation (≥0.907) and validation (≥1) sets were 0.067 and 0.060, respectively. The regression coefficients of the lowest and highest quartile groups were not equal overall (p=0.021). In both data sets, the mean predicted value was overestimated in the lowest quartile group, but underestimated in the highest quartile group.

**Table 5:** Performance in Model 2 according to EQ-5D quartile in the derivation and validation sets

	**Derivation set**		**Validation set**	
The lowest quartile				
N	224		33	
RMSE (% RMSE)	0.137 (11.7)		0.119 (10.2)	
MAE (SD)	0.100 (0.093)		0.060 (0.057)	
EQ-5D index	Actual	Predict	Actual	Predict
Mean (SD)	0.647 (0.125)	0.726 (0.093)	0.723 (0.070)	0.806 (0.086)
The highest quartile				
N	267		37	
RMSE (% RMSE)	0.087 (8.7)		0.003 (0.27)	
MAE (SD)	0.067 (0.055)		0.060 (0.049)	
EQ-5D index	Actual	Predict	Actual	Predict
Mean (SD)	0.960 (0.045)	0.902 (0.057)	1.000(0.000)	0.940 (0.049)

## Discussion

This study explored an algorithm for mapping the EORTC QLQ-C30 onto the EQ-5D index. Model 2, which included global health status, physical functioning, role functioning, emotional functioning, and pain as explanatory variables, was preferred over the full model, due to its predictability, logical consistency, and parsimony. Although mapping the EORTC QLQ-C30 onto the EQ-5D index has been assessed previously, those studies evaluated patients with specific types of cancer, including gastric [5], esophageal [13], and breast [12,14] cancers and multiple myeloma [19]. By contrast, the present study evaluated patients with 28 different types of cancer, providing our mapping model with the advantage (over earlier mapping algorithms) of being applicable to all cancer patients in Korea.

We also explored models using only functional scales as explanatory variables and a backward elimination model of the functional scale (data not shown). We found that Model 2 showed optimum performance, retaining the global health, physical, role, emotional functioning, and pain scales. The MAE of this model, 0.066, was lower than the MAE of 0.092 reported in another Korean study [14]. The adjusted R^2^ of our derivation set was 0.516 and the normalized RMSE was 8.1%. We also analyzed our data based on the UK tariff [20] using backward elimination regression. Although the remaining variables were the same as those using the Korean tariff, the magnitude of the absolute coefficients increased. The MAE of the UK backward elimination model for our derivation set was 0.156, and the adjusted R^2^ was 0.463. A systematic review reported that R^2^ statistics for condition-specific measures relative to generic measures generally ranged from 0.4–0.6 [18]. Another study showed that the backward regression model resulted in better predictability than the full model, with the former showing an adjusted R^2^ of 0.8 and a normalized RMSE of 6.02% for the derivation set; that study, however, included squared terms, such as the square of the physical functioning scale [12]. Use of OLS with a stepwise regression model retaining three scales (global health, physical, and emotional functioning) yielded an adjusted R^2^ of 0.611 and a normalized RMSE of 12.0% for the derivation set [5]. Application of OLS regression using all of the scale scores in patients with esophageal cancer resulted in variables slightly different from those previously reported, including global health, role, emotional, cognitive function, pain, and fatigue, with an adjusted R^2^ of 0.611 for the derivation set [13].

OLS regression tends to overestimate the true value of EQ-5D utilities for patients in poor health, while underestimating the true EQ-5D utilities at the upper end of the scale [14,21,22]. Our model showed the same trend, overpredicting the mean EQ-5D index in the group of patients in relatively poor health. The MAE of the best performing model increased from 0.056 in the relatively healthy group to 0.078 in the group with relatively poor health. Caution is therefore warranted when applying a mapping function to patients in poor health, and further research is needed regarding the cut-off points for the use of the EORTC QLQ-C30 on patients in poor health. Mapping is the second best alternative to the direct use of a preference-based measure because mapped estimates can yield large errors, particularly when mapping from condition-specific to generic preference-based measures [18]. This, however, may not be as important for QLQ C-30 mappings.

The mapping algorithm formulated in this study may have limited generalizability, because the participants in the validation set came from only one hospital. Although our sample included individuals with various conditions, further research with samples from other institutions would be helpful.

## Conclusions

The mapping model using OLS regression showed a reasonable predictive ability. This mapping algorithm may enable researchers to convert results from the EORTC QLQ-C30 to the EQ-5D utility indexes for Korean cancer patients. Nevertheless, using OLS regression to predict very low and high EQ-5D indices remains challenging. These findings may help when assessing the performance of cost-utility analyses of the use of healthcare interventions in cancer patients.

## Abbreviations

EORTC QLQ-C30: European Organisation for Research and Treatment of Cancer Quality of Life Questionnaire Core 30; HRQoL: Health-related quality of life; RMSE: Root-mean-squared error; MAE: Mean absolute error; OLS: Ordinary least squares.

## Competing interests

The authors have no disclosures.

## Authors’ contributions

All authors contributed to the conception and design of the study, the acquisition of data, and the interpretation of the results. SHK analyzed the data and was involved in drafting the manuscript; MWJ, HJK and JHA were involved in revising the manuscript to ensure its critically important content. All authors have read and approved the final manuscript.
